# Financial Impacts of Liver Fluke on Livestock Farms Under Climate Change–A Farm Level Assessment

**DOI:** 10.3389/fvets.2020.564795

**Published:** 2020-12-07

**Authors:** Shailesh Shrestha, Alyson Barratt, Naomi J. Fox, Bouda Vosough Ahmadi, Mike R. Hutchings

**Affiliations:** ^1^Department of Rural Economy, Environment and Society, Scotland's Rural College (SRUC), Edinburgh, United Kingdom; ^2^Animal and Veterinary Sciences, Scotland's Rural College (SRUC), Edinburgh, United Kingdom; ^3^The European Commission for the Control of Foot and Mouth Disease (EuFMD), Animal Production and Health Division (AGAH), Food and Agricultural Organization (FAO), Rome, Italy

**Keywords:** climate change, liver fluke, livestock farms, farm level modeling, economic impact study

## Abstract

Liver fluke infection (fascioliasis) is a parasitic disease which affects the health and welfare of ruminants. It is a concern for the livestock industry and is considered as a growing threat to the industry because changing climatic conditions are projected to be more favorable to increased frequency and intensity of liver fluke outbreaks. Recent reports highlighted that the incidence and geographic range of liver fluke has increased in the UK over the last decade and estimated to increase the average risk of liver fluke in the UK due to increasing temperature and rainfall. This paper explores financial impacts of the disease with and without climate change effects on Scottish livestock farms using a farm-level economic model. The model is based on farming system analysis and uses linear programming technique to maximize farm net profit within farm resources. Farm level data from a sample of 160 Scottish livestock farms is used under a no disease baseline scenario and two disease scenarios (with and without climate change). These two disease scenarios are compared with the baseline scenario to estimate the financial impact of the disease at farm levels. The results suggest a 12% reduction in net profit on an average dairy farm compared to 6% reduction on an average beef farm under standard disease conditions. The losses increase by 2-fold on a dairy farm and 6-fold on a beef farm when climate change effects are included with disease conditions on farms. There is a large variability within farm groups with profitable farms incurring relatively lesser economic losses than non-profitable farms. There is a substantial increase in number of vulnerable farms both in dairy (+20%) and beef farms (+27%) under the disease alongside climate change conditions.

## Introduction

Liver fluke (fascioliasis) is a parasitic disease caused by *Fasciola hepatica* and is distributed globally (1). The disease is a concern for livestock industry both from an animal health perspective but also due to the economic consequences on production systems. The economic impact is caused by lower production due to reduced body weight, milk yield and fertility as well as health conditions such as diarrhea and mortality in cattle (2). It is estimated to cost the livestock sector around £2 billion per year globally (3, 4). Liver fluke is endemic in the United Kingdom costing the cattle industry between £13 and £40 million annually (5) and more recent estimate is £31 million per year.

Liver fluke has a complex lifecycle with definitive mammalian hosts (primarily cattle and sheep), a number of free-living stages in the environment, and an intermediate molluscan host *Galba truncatula* (a species of pond snail). The parasite's free-living stages thrive in warm and wet conditions which also promote the survival and reproduction of *G. truncatula*. Due to the influence of temperature and moisture on multiple stages of this lifecycle, changing climatic conditions influence the timing, intensity, and distribution of fascioliasis outbreaks. The dynamics of livestock parasites and hence the disease patterns are shifting under climate change with larger spread and more frequent outbreaks of disease (2, 6–9). For example, liver fluke outbreaks were historically restricted to the south west of the UK, but it is spreading to other regions especially north of the UK more recently (10, 11). The future climatic conditions are projected to increase liver fluke incidences on livestock farms in the UK (12). Predictions of long-term fluke risk indicate that future risk is greater than past risk across the UK, and some areas are set to experience unprecedented epidemics over the next 60 years (12). An ex-ante study, hence, to estimate economic losses from the disease at production level under future climate is essential to control and minimize the impacts of this disease. It can be used to highlight the importance of making long term decisions to control the disease and minimize such risks in advance. Several studies have suggested that different farms respond differently to changed farm conditions (such as policy changes or disease outbreaks) due to the variabilities present between them (13–17). It is hence essential to look at the impacts of the disease at farm level to determine where the impacts would be greater. This paper aimed to determine the disease impacts on farms taking account of farm variability. The variability was based on production systems (dairy and beef production systems) and on farm profitability. There are substantial differences in profitability between farms in Scotland where many farms rely heavily on farm support payments to stay profitable (18). This makes them vulnerable to any type of changes in support payment policies especially in recent times when the UK agricultural policies are undergoing some significant changes bringing in uncertainties associated with support payments in future (17, 19). This paper used farms' reliance on farm support payments to be an indicator of farm economic vulnerability and explored changes in the number of vulnerable farms under the disease scenarios. A dynamic optimizing farm level model, ScotFarm was implemented for this study which used farm net profit as a measure to determine the economic impact of the disease on dairy and beef farms. This paper contributes to improve our understanding on long term economic impacts of liver fluke disease on Scottish livestock farms with and without future climate change conditions at a farm level.

## Methodology

### ScotFarm Model

ScotFarm is a farm-level linear programming model that optimizes financial margins of a farm within its bio-physical constraints (20). This model has been used in a number of earlier studies (21–24). The model maximizes farm net profit which is the sum of gross revenues from all farm activities and farm support payments such as Basic Payments Scheme and Less Favorable Area Scheme (25, 26), minus fixed costs (FC). Farm net profit is measure that is used widely to measure farm's financial performance (18, 19, 27). The general mathematical formulation of maximizing farm net profit was as follows:

$$\begin{array}{l} Max\;Z=\sum_{i}^{\;}[grev_{i}]x_{i}+FS-FC\;\forall f \end{array}$$

Subject to

$$\begin{array}{l} \underset{i}{\sum }A_{i}x_{i}\leq b_{f}\;;\;x_{i}\;\geq \;0\;\forall f \end{array}$$

Where *Z* denotes maximized net profit of all activities from all the enterprises of a farm; *grev* represents gross revenue of an enterprise; index *i* denotes agricultural activities including livestock and crop while *f* denotes individual farms; *x*_,*i*_ is the non-negative activity level in hectares or heads of farm *f* activity *i*; *FS* represents all support payment received by a farm; *FC* is total fixed costs; *A* is an input–output coefficient for activity *x*; and *b* denotes limited farm resources.

Gross revenue of an enterprise (*grev*_*i*_) was estimated as follows:

$$\begin{array}{l} grev_{i}=\sum {p_{i,j}y}_{i,j}-\;CR_{i}-\sum VC_{i}-\sum NC_{i}\;\forall f \end{array}$$

Where *p* denotes price of output *j, y* is the quantity of output *j per activity x*_, *i*_; *CR* denotes the cost of replacement; *VC* represents variable costs (including labor, veterinary and AI costs) and *NC* represents feed costs which includes purchased concentrate and grass silage.

The model consisted of livestock component (representing dairy or beef production systems) which were constraint over limiting resources such as land, labor, feed, and replacement stocks. These limiting resources (except for land which was fixed) could be brought from external sources if farm's own resources were not sufficient to carry out farm activities. Labor used on farm was determined by balancing out labor requirement default values (28) for each of the animals on farm and total labor available on farm (i.e., family labor and hired labor if required). Similarly, total feed used on farm was determined based on energy and protein requirement of each of the animals on farm, feed produced on farm (grass, grass silage and grain silage) and feed (grass silage and concentrate feed) from external sources if required. The model assumed a 4-year production cycle for all livestock systems, where a minimum of 25% of animals were culled each year and replaced by either own farm-produced or bought-in replacements. Animal number on a particular year on farm was based on animal number on the previous year, culled animals and replaced animals. To determine calf numbers calving and mortality rates were included.

For dairy farms, total milk production was the summation of milk produced by all lactating cows and assumed to be sold to the market. There was no consideration for spillage, discards, or own consumption. All male calves born on farm were sold and considered as another output for dairy farms. For beef farms, the main outputs were calves, 18-month beef, 24-month beef and lambs (if the farm has a sheep production activity). Farm resources such as labor and feed required to produce these outputs were determined based on number of animals on farm each year.

The model was run under three scenarios; a baseline scenario *(“baseline”)* where farms were assumed to have disease-free production system and two disease scenarios; (i) a standard disease scenario *(“disease”)* with an assumption that a farm production system was under a standard prevalence rate of liver fluke and (ii) a climate change diseased scenario *(“disease*+*cc”)* where it was assumed that a farm had a “diseased production system” under climate change conditions. The farm net profit and production level under both disease scenarios were compared with corresponding outputs under the baseline scenario to determine the impact of the disease on farms.

### Data Input

#### Farm Level Data

Farm level data were taken from the Scottish Farm Business Survey (FBS), a survey conducted annually in Scotland (18). The FBS collects physical and economic farm level data in a sample of around 550 representative farms each year. The sample data used in this paper contained 50 dairy farms and 110 beef farms which were studied separately to analyse economic impact of the disease between those two livestock production systems. The farm variability within a production system was considered by using farm profitability, where a comparison of the highest profit-making farms (top 25% farms) and the lowest profit making (bottom 25% of farms) was undertaken. Farm vulnerability (*v*_*f*_) was determined using the ratio of farm support payment (*S*_*f*_) on farm net profit (ρ_*f*_). For this study, a farm (*f*) was considered vulnerable if total support payment (*S*_*f*_) it received was higher than net farm profit, such as;

$$\begin{array}{l} v_{f}\;if\frac{\;\rho_{f}}{S_{f}}<1 \end{array}$$

#### Disease Parameters

The disease parameters (Table 1) used under the standard “*disease*” scenario for this study were taken from a Herd Partial Budget model (29) and a National Welfare model. The disease prevalence on dairy farms was estimated slightly higher (19.3%) than that on beef farms (13%). In the model, the loss in production and direct cost per infected animals were determined at UK-wide dairy and beef production levels. Loss in production included reduction in milk yield in case of dairy and reduction in carcass weight in case of beef animals. An increase in 1% of culling rate was also included in the model. The direct costs included veterinary and medicine costs and added to the variable costs of each of the infected animals.

**Table 1:** Disease parameters used in the model.

**Parameters**	**Dairy**	**Beef**
Disease prevalence^a^	19.3%	13%
Loss in production^a^	7.7%	0.5%
Direct cost (£/infected animal)^b^	86.15	20.40

*Source: ^a^6; ^b^30*.

#### Climate Change Parameters

The climate change “*disease*+*CC*” scenario used the A1B^1^ emission scenario which was a part of UKCP09 using HadRM3 model (31). Disease prevalence under the climate change scenario was based on an earlier study (12) which used the Ollernshaw index to estimate disease risk in the UK under climate change. This disease risk under climate change scenario (a 50% increase in prevalence) was used as a proxy for disease prevalence under climate change in this paper (Table 2). This scenario includes climate change effects not only on the disease but also on the production system directly affecting individual animals. Two parameters, changes in grass production and loss in production due to heat stress were assumed to be the changes that affected individual production level of an animal under climate change. Grass yield change parameter was taken from our earlier study (32) which used a dynamic crop model, COUP (32) to simulate grass growth under climate change scenario. The production loss parameter due to heat stress was based on a study in the UK which looked at impact of heat stress on livestock farms (33). In addition to that, a small increase in direct variable costs under climate change was assumed (32). The additional direct costs include small adjustments made on farms to minimize heat stress such as providing additional water and increase in veterinary care.

**Table 2:** Change parameters used in the model under the “*disease*+*CC*” scenario compared to the *baseline* scenario.

**Parameters**	**Change**
Prevalence	+50%^a^
Grass production	+35%^b^
Direct costs	+10%^b^
Loss in production	−6%^c^

*Source: ^a^13; ^b^32; ^c^33*.

## Results

### Farm Variability

There was a significant difference between dairy and beef farms both in physical and economic terms (Table 3). On average Scottish dairy farms were significantly larger in terms of farm area, herd size, fixed costs and farm net profit than Scottish beef farms. Beef farms, however, received higher farm support payments, which was almost three times higher than the farm net profit indicating a significant reliance on farm support payment.

**Table 3:** Average farm variables on Scottish dairy and beef farms (st. dev. in parenthesis).

**Farm variable**	**Dairy *n* = 50**	**Beef *n* = 110**
Arable land (ha)	15.5 (21.0)	7.5 (12.9)
Grass land (ha)^*^	143.2 (72.0)	120.9 (78.2)
Dairy/Beef Herd size (LU^†^)^**^	321 (151)	161 (108)
Sheep herd size (LU^†^)^*^	7 (10)	24 (11)
Family labor (hrs)	3,582 (1317)	3,124 (1068)
Stocking rate^**^ (LU^†^/ha)	2.14 (0.8)	1.33 (0.5)
Milk yield (ltr/cow)	7,207 (1668)	na
Farm support payment^a^ (£)^**^	38,011 (21,575)	54,993 (30,352)
Variable costs (£/cow)	240 (75)	245 (74)
Fixed costs (£)^**^	129,098 (61,880)	57,349 (91,773)
Farm net profit (£)^*^	40,468 (90,550)	29,018 (38,869)
Support payment share^**^	0.93 (1.61)	2.91 (9.8)

a Farm support consists of direct farm payments and agri-environment scheme payments.

Significant difference between dairy and beef farm variable at

* P < 0.05;

** P < 0.01 levels;

† *LU= livestock unit (34)*.

### Farm Net Profit

**Figure 1:**
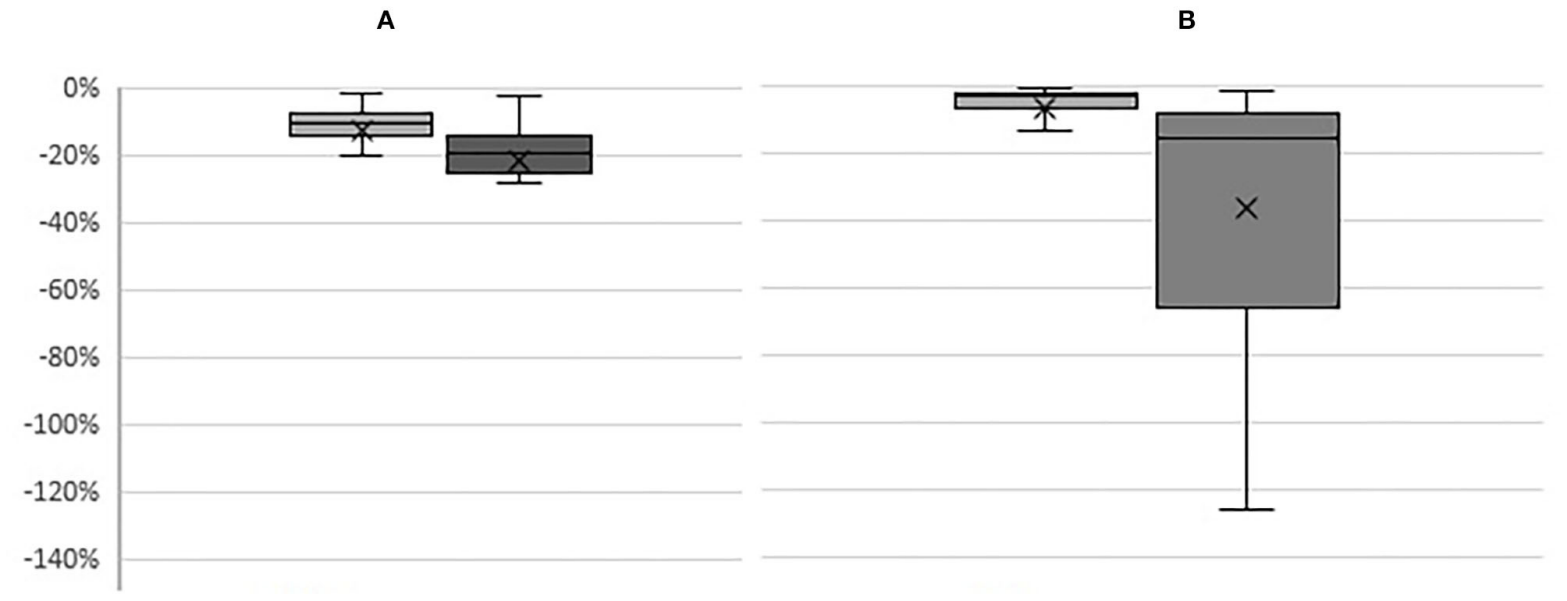
Boxplots representing percentage changes in net profit on **(A)** dairy and **(B)** beef farms under “disease” 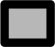 and “disease + cc” 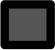 scenarios compared to the baseline scenario; (line is the median and cross is the average value for each farm groups; y-axis represents percentage).

All sampled farms showed reduction in farm net profits under the standard “*disease*” scenario (light colored boxes in Figure 1). There was a small difference in the impact of disease on dairy and beef production systems with beef farms projected to have a smaller loss with an average reduction of 6% in farm net profit compared to dairy farms which were projected to lose on average 12%.

There was, however, substantial increase in loss especially on beef farms when climate change effects are included. Under the “*disease*+*CC*” scenario (dark colored boxes in Figure 1), the average loss on dairy farms was projected to increase up to 24% and on beef farms up to 36%. There was also a higher variation of disease impact under the “*disease*+*CC”* scenario in beef farm group compared to that in dairy farm group.

The impact of disease on farm net profit was different between profitable and non-profitable farms. The differences in impacts of the disease between the profitable farms (Top quarter farms) and the non-profitable farms (Bottom quarter farms) were highly significant under both of the “*disease”* and “*desease*+*CC”* scenarios (Table 4). The top and bottom performing farms within beef farms, however, only showed a significance in difference in impacts of disease under the “*disease*+*CC”* scenario but not under the “*disease”* scenario.

**Table 4:** Percentage changes in farm net profit on farms in the top quarter and bottom quarter of dairy and beef farm groups compared to the baseline scenario.

**Farm type/scenarios**	**Top quarter farms**	**Bottom quarter farms**
Dairy		
disease^**^	−7%	−23%
disease+CC^**^	−11%	−37%
Beef		
*disease*	−4%	−7%
disease+CC^**^	−5%	−95%

** *P < 0.01*.

### Farm Production

**Figure 2:**
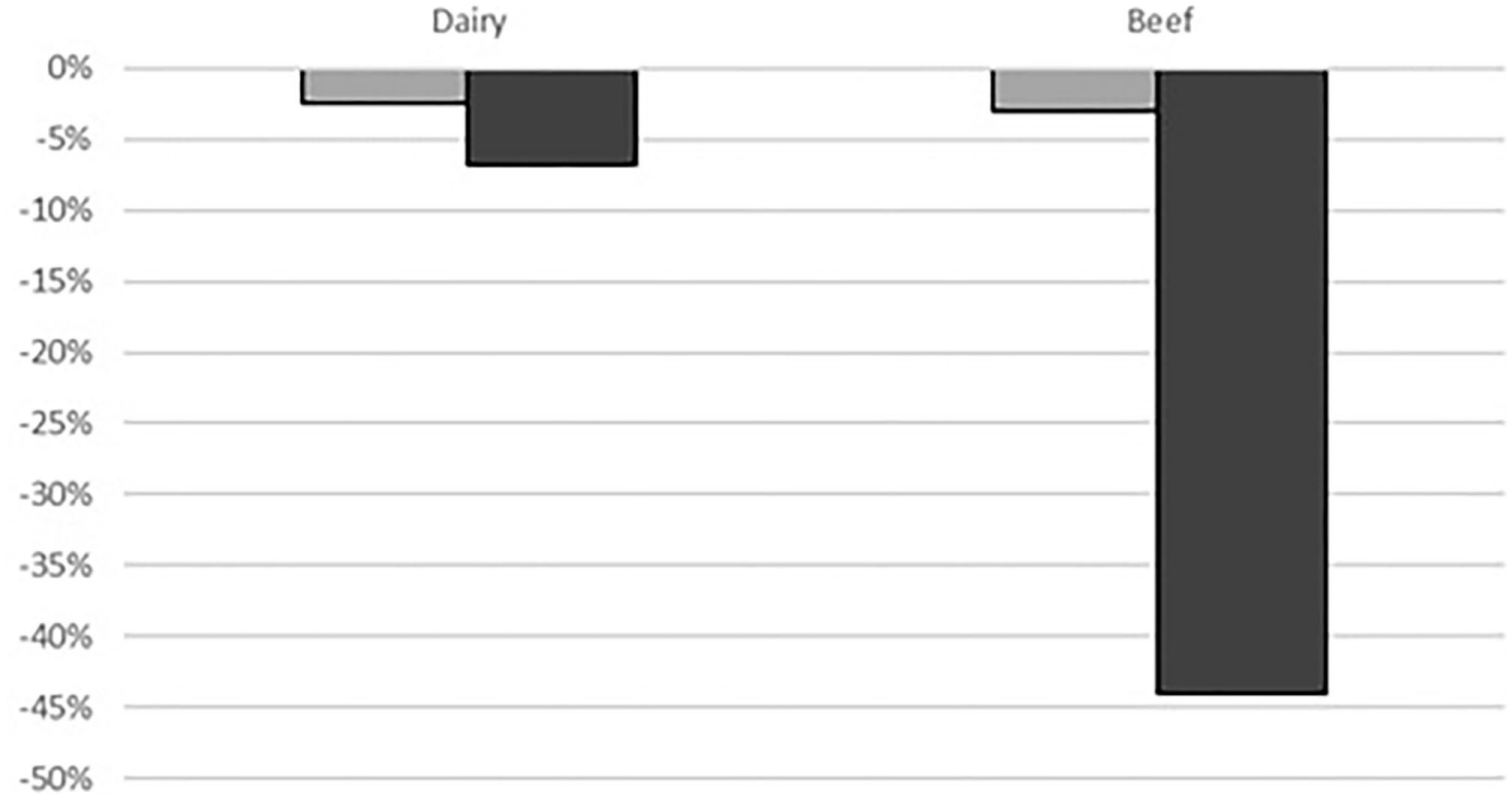
Percentage change in animal numbers on dairy and beef farms under 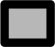 “*disease*” and 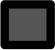 “*disease*+*CC*” scenarios compared to the baseline scenario (y-axis represents percentage).

In this analysis, change in livestock numbers on farms (number of lactating cows for dairy and number of suckler cows for beef farms) is assumed to be representing change in farm production. Dairy farms were projected to reduce their production by 2.5% under the standard “*disease*” scenario and by 7% under the “*disease*+*CC*” scenario (Figure 2). Beef farms were expected to reduce animal numbers by 3% under the standard “*disease*” scenario but a substantial reduction was expected (44%) under the “*disease*+*CC*” scenario.

### Farm Vulnerability

**Figure 3:**
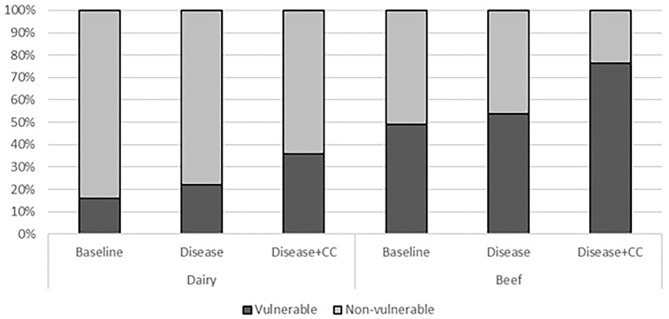
Proportion of vulnerable and non-vulnerable dairy and beef farms (y-axis represents percentage).

As shown in Figure 3, there were fewer dairy farms (16%) in the “vulnerable” category farms compared to those in beef farms (48%) in the baseline scenario. Under the “*disease*” scenario, there was a small increase in percentage of the number of vulnerable farms for both dairy (+6%) and beef (+3%) farm types compared to the baseline scenario. However, there was a substantial increase in percentage of vulnerable farms for both dairy farms (+20%) and beef farms (+27%) under the “*disease*+*CC*” scenario. This resulted in more than one-third of total dairy farms and three-fourths of total beef farms in the “vulnerable” category of the farms.

## Discussion and Conclusions

Liver fluke infection has economic consequences on a livestock production system and due to unequivocal assumptions of future climatic conditions to be more favorable for the disease to flourish, we included climate change effects to examine the economic impact of the disease at a farm level production system. This means, our analysis not only included the effect of climate change on disease prevalence alone but also changes in grass production and livestock production due to heat stress.

The analysis shows a reduction in farm net profit of 12% on an average dairy farm and 6% on an average beef farm on a standard disease scenario. This reduction in profit increases by 2-folds for dairy farms and by 6-folds for beef farms when climate change effects are included. This highlights the importance of including climate change effects in ex-ante economic impact studies of liver-fluke disease. The difference in impact between dairy and beef farming systems is due to the variability in farm management and productivity in those two faming systems. An average dairy farm in Scotland is considered to be more efficient compared to an average beef farm (18). The impact of disease without climate change effects is lesser on an average beef farm compared to that on an average dairy farm due to a lower prevalence rate of disease, a smaller loss in production and a relatively lower average farm net profit on a beef farm. Higher disease prevalence for dairy farms compared to beef farms is due to the difference in location and farm management. Scottish dairy farms are mostly located in south west region which reported higher incidence of liver fluke than other regions (12). In addition, the Scottish beef farms have a more extensive production system than dairy farms which also contributes to a lower disease prevalence.

The impact of disease on beef farms increase substantially under climate change compared to that on dairy farms. Although, the climate change parameters are assumed to be same as in dairy production systems, an increase in marginal cost (due to increased variable cost) and decrease in marginal profit (due to larger loss in production) under climate change play a significant role in unbalancing farm net profit on beef farms. In the context of climate change, for almost half of the sampled beef farms, costs due to the disease make beef production unprofitable, leading farmers to substantially decrease their production. Some of these farms (41%) decreased their production to minimize losses. There was a small gain from increased grass production under climate change which was offset by the lower feed intake due to heat stress.

There is also a significant difference in the impact of the disease both with and without climate change effects between farms in the top quarter and bottom quarter in dairy farm group. It clearly highlights the importance of including farm variability within a farm type to conduct an impact assessment of farms. Many earlier farm level impact assessment studies analyzing economic impacts of external shocks such as change in policy and market prices presented similar conclusions (13, 14, 21). Within dairy farm types, the farms in the top quarter are more efficient producers and have higher profits than farms in the bottom quarter. Those farms have higher yielding animals, higher productivity and also receive higher price for their products. Although the disease effects were similar on those farms as to other farms, those farms are efficient farms and more capable of adjusting their systems (such as by purchasing less concentrates) to minimize the impact of the disease. The relative reduction in net profit due to disease on farms in the top quarter is hence smaller. Unlike dairy farms, there is a very small variability in impact of disease between beef farms under the standard “disease” scenario. Most of beef farms have smaller profits and the difference in profitability between farms in the top and bottom quarter is small, hence show relatively small variability in impact of disease. However, under “*disease*+*CC*” scenario, there is a substantial increase in variability in impact of disease between beef farms. Beef farms in the bottom quarter reduce their production significantly. These farms have larger reductions in net profit and the difference in impact of disease on these farms compared to farms in the top quarter becomes very significant.

There are almost half of beef farms that rely on farm support to be profitable (vulnerable farms) compared to only 16% of dairy farm with such vulnerability. The impact of liver-fluke on vulnerability is almost similar on both farm types. However, when climate change effects are considered, the disease increases vulnerable farms by 27% in beef farm group and 20% in dairy farm group. This means adding climate change effects to disease would substantially increase number of vulnerable farms in livestock production system.

The results and analysis presented in this paper are solely based on disease impact at farm level. The economic consequences of the disease on a livestock farm were assumed to be due to loss in production and increase in variable costs in this study. It should be noted that the disease has wider economic implications beyond the farm gate such as changes in market prices (35) due to reduced supply which might have additional effects on livestock farms.

## Data Availability Statement

The original contributions generated for this study are included in the article/supplementary materials, further inquiries can be directed to the corresponding author/s.

## Author Contributions

SS is the main author of the paper. AB contributed in running the model and writing and analysis of the results. NF contributed in liver fluke prevalence work and writing and analysis of the results. BV contributed in result analysis and writing the paper. MH contributed in writing the paper and providing support to conduct this work. All authors contributed to the article and approved the submitted version.

## Conflict of Interest

The authors declare that the research was conducted in the absence of any commercial or financial relationships that could be construed as a potential conflict of interest. The reviewer JN declared a past co-authorship with one of the authors BV to the handling Editor.

## Footnotes

^1^A balanced emission scenario as defined by Intergovernmental Panel on Climate Change (30).

## References

[1] Rojo-Vázquez F Meana A Valcárcel F Martínez-Valladares M Update on trematode infections in sheep. Vet Parasitol. (2012) 189:15–38. 10.1016/j.vetpar.2012.03.029 22521973

[2] Skuce P Zadoks R Liver fluke–a growing threat to UK livestock production. Cattle Pract. (2013) 21:138–49. Available online at: https://www.bcva.org.uk/system/files/whatwedo/Sample%20CP%20paper.pdf (accessed March 21, 2020).

[3] FAO Diseases of Domestic Animals Caused by Liver Flukes: Epidemiology, Diagnosis and Control of Fasciola, Paramphistome, Dicroceoelium, Eurytrema and Schistosome Infections of Ruminants in Developing Countries. Food and Agricultural Organization of the United Nations report (1994).

[4] Piedrafita D Spithill TW Smith RE Raadsma HW. Improving animal and human health through understanding liver fluke immunology. Parasite Immunol. (2010) 32:572–81. 10.1111/j.1365-3024.2010.01223.x 20626812

[5] Bennett R Ijpelaar J. Economic Assessment of Livestock Diseases in Great Britain. Final Report to the Department for Environment, Food and Rural Affair (2003).10327436

[6] Gale P Drew T Phipps LP David G Wooldridge M. The effect of climate change on the occurrence and prevalence of livestock diseases in Great Britain: a review. J Appl Microbiol. (2009) 106:1409–23. 10.1111/j.1365-2672.2008.04036.x 19191974 PMC7197753

[7] Gauly M Bolwein H Breves G Brügemann K Dänicke S Daş G. Future consequences and challenges for dairy cow production systems arising from climate change in Central Europe – a review. Switzerland: Zurish Open Repository and Archive, University of Zurich (2013). 10.1017/S1751731112002352 23253935

[8] Fox NJ Davidson RS Marion G Hutchings MR. Modelling livestock parasite risk under climate change. Adv Anim Biosci. (2015) 6:32–4. 10.1017/S204047001400048X26486780

[9] Fox NJ Marion G Davidson RS White PC Hutchings MR. Climate-driven tipping-points could lead to sudden, high-intensity parasite outbreaks. R Soc Open Sci. (2015) 2:140296. 10.1098/rsos.140296 26064647 PMC4453250

[10] Kenyon F Sargison ND Skuce PJ Jackson F. Sheep helminth parasitic disease in south eastern Scotland arising as a possible consequence of climate change. Vet Parasitol. (2009) 163:293–7. 10.1016/j.vetpar.2009.03.027 19556065

[11] Pritchard GC Forbes AB Williams DJL Salima-Bejestani MR Daniel RG. Emergence of Fasciolosis in cattle in East Anglia. Vet Rec. (2005) 157:578–82. 10.1136/vr.157.19.578 16272544

[12] Fox NJ White PC McClean CJ Marion G Evans A Hutchings MR. Predicting impacts of climate change on *Fasciola hepatica* risk. PLoS ONE. (2011) 6:e16126. 10.1371/journal.pone.0016126 21249228 PMC3018428

[13] Ramsden S Gibbons J Wilson P Impacts of changing relative prices on farm level dairy production in the UK. Agric Syst. (1999) 62:201–15. 10.1016/S0308-521X(99)00065-7

[14] Shrestha S Hennessy T Hynes S The effect of decoupling on farming in Ireland: a regional analysis. Irish J Agric Food Res. (2007) 46:1–13. Available online at: https://www.jstor.org/stable/25564551.

[15] Hennessy T Shrestha S Farrell M Quantifying the viability of farming in Ireland: can decoupling address the regional imbalances? Ir Geogr. (2008) 41:29–47. 10.1080/00750770801909342

[16] Acs S Hanley N Dallimer M Gaston KJ Robertson P Wilson P The effect of decoupling on marginal agricultural systems: implications for farm incomes, land use and upland ecology. Land Use Policy. (2010) 27:550–63. 10.1016/j.landusepol.2009.07.009

[17] Shrestha S Thomson S Ahmadi BV Barnes A Assessing the impacts of alternative post-Brexit trade and agricultural support policy scenarios on Scottish farming systems. (2018). REES, SRUC. Available online at: https://www.sruc.ac.uk/downloads/file/3606/assessing_the_impacts_of_alternative_post-brexit_trade_and_agricultural_support_policy_scenarios_on_scottish_farming_systems (accessed March 22, 2020).

[18] Scottish Government Scottish Farm Business Income: Annual Estimates 2017/18. (2019). Available online at: https://www.gov.scot/publications/scottish-farm-business-income-estimates-2017-18/ (accessed February 20, 2020).

[19] Hubbard C Davis J Fend S Harvey D Liddon A Moxey A Brexit: how will UK agriculture fare? EuroChoices. (2018) 17:19–26. 10.1111/1746-692X.12199

[20] Shrestha S ScotFarm – A Farm Level Optimising Model. (2018). Available online at: https://www.sruc.ac.uk/downloads/file/4740/scotfarm_manual (accessed January 02, 2020).

[21] Ahmadi BV Shrestha S Thomson SG Barnes AP Stott AW Impacts of greening measures and flat rate regional payments of the Common Agricultural Policy on Scottish beef and sheep farms. J Agric Sci. (2015) 153:676–88. 10.1017/S0021859614001221

[22] Eory E MacLeod M Shrestha S Roberts D Linking an economic and a biophysical model to support farm GHG mitigation policy. Ger J Agric Econ. (2014) 63:133–42. Available online at: https://www.gjae-online.de/articles/linking-an-economic-and-a-life-cycle-analysis-biophysical-model-to-support-agricultural-greenhouse-gas-mitigation-policy/ (accessed January 05, 2020).

[23] Glenk K Shrestha S Topp K Sanchez B Iglesias A Dibari C A farm level approach to explore farm gross margin effects of soil organic carbon management. Agric Syst. (2017) 151:33–46. 10.1016/j.agsy.2016.11.002

[24] Shrestha S Ahmadi BV Barratt A Thomson S Stott A. Financial vulnerability of dairy farms challenged by Johne's disease to changes in farm payment support. Front Vet Sci. (2018) 5:316. 10.3389/fvets.2018.00316 30619898 PMC6305583

[25] Scottish Government Basic Payment Scheme. Rural Payments and Services (2018). Available online at: https://www.ruralpayments.org/topics/all-schemes/basic-payment-scheme/ (accessed February 20, 2020).

[26] Scottish Government Less Favoured Area Support Scheme. Rural Payments and Services (2018). Available online at: https://www.ruralpayments.org/topics/all-schemes/lfass/ (accessed February 20, 2020).

[27] DEFRA Farm Business Income by types of farm in England, 2018/19. (2019). Available online at: https://assets.publishing.service.gov.uk/government/uploads/system/uploads/attachment_data/file/847722/fbs-businessincome-statsnotice-21nov19.pdf (accessed February 21, 2020).

[28] FMH Farm Management Handbook 2016/17. UK: SAC Consultancy (2017).

[29] Tongue SC Correia-Gomes C Eze JI Henry MK Stott AW Milne CE Liver fluke – Fasciola hepatica: comparative losses in key sectors of the British cattle industry. In: Proceedings of the 14th symposium of the International Society for Veterinary Epidemiology and Economics. Merida, Mexico (2015).

[30] IPCC IPCC Special Report: Emissions scenarios - Summary for Policymakers. (2000). Available online at: https://www.ipcc.ch/site/assets/uploads/2018/03/sres-en.pdf (accessed August 10, 2019).

[31] MetOffice. UK Climate Projections: Briefing Report. (2010). Available online at: https://webarchive.nationalarchives.gov.uk/20181204111026/http://ukclimateprojections-ukcp09.metoffice.gov.uk/22530 (accessed August 12, 2019).

[32] Shrestha S Tarsitano D Topp K Eory V Adaptation to climate change at farm level – Scottish beef farms. In: *Paper presented at the 3rd European Climate Change Adaptation Conference, Glasgow 5th-9th June, 2017*. (2017).

[33] Fodor N Foskolos A Topp CFE Moorby JM Pasztor L Foyer CH. Spatially explicit estimation of heat stress-related impacts of climate change on the milk production of dairy cows in the United Kingdom. PLoS ONE. (2018) 13:e0197076. 10.1371/journal.pone.0197076 29738581 PMC5940184

[34] FBS Farm Business Survey Datasets 2016-17. Scottish Government (2017). Available online at: https://www2.gov.scot/Topics/Statistics/Browse/Agriculture-Fisheries/Publications/FASdata (accessed February 20, 2020).

[35] Barratt AS Arnoult MH Ahmadi BV Rich KM Gunn GJ Stott AW A framework for estimating society's economic welfare following the introduction of an animal disease: the case of Johne's disease. PLoS ONE. (2018) 13:e0198436 10.1371/journal.pone.0198436 29874292 PMC5991423

